# A Resident-Led Quality Improvement Project in a Community Based Hospital Emergency Department – The Benefits of Simplified Plan-Do-Study-Act/Patient-Safety Quality Improvement Projects Regardless of Staffing Levels

**DOI:** 10.51894/001c.123236

**Published:** 2024-09-09

**Authors:** Martina Ghiardi, Shauncie Skidmore, Christina George, Rachael Crise, Olga J. Santiago

**Affiliations:** 1 Emergency Department McLaren Oakland Hospital, Pontiac, Michigan; 2 Independent Consultant, Clarkston, Michigan; 3 Convenant Medical Center Cooper Hospital, Saginaw, Michigan; 4 Ascension St. Joseph Hospital, Tawas City, Michigan; 5 McLaren Oakland Hospital, Pontiac, Michigan

**Keywords:** Emergency department, Patient safety, Quality improvement, Simple lacerations repair

## Abstract

**Introduction:**

An emergency department (ED) resident believed ED patients, who needed a simple laceration repair, would be better served if the ED used a laceration cart for supplies, as opposed to the hunt-and-gather method for collecting needed supplies. To address this issue, a two-step Plan-Do-Study-Act/Patient-Safety quality improvement (PDSA/PS QI) project was initiated, with the intent that the project could be completed in a timely manner regardless of staffing levels. The primary purpose of the project was two-fold: 1) to explore the possible time-to-repair benefits of using a laceration repair supply cart in the emergency department and 2) to determine the feasibility of conducting a simple multi-cycle PDSA/PS QI project in a potential staffing-shortage environment.

**Methods:**

A prospective study using a simple 2-cycle PDSA/PS QI procedure was initiated. During cycle 1, baseline data, to determine the time to complete simple-laceration repairs using a hunt-and-gather supply process, was collected in the form of sign-out/return sheets located next to a laceration repair kit. Cycle 2 introduced the use of a simple-laceration supply cart in the ED, with data collected in the form of a sign-out/return sheet located on the supply cart. Data analysis included a two-sample Wilcoxon rank-sum (Mann-Whitney) test to assess the effectiveness of the suture cart implementation.

**Results:**

Pre-intervention. Twelve valid cases were recorded on the sign-out/return sheets. The baseline time range to complete a simple laceration repair varied from 26 minutes to 151 minutes, with an average of 68.3 minutes (SD=40.8).

Post-intervention. Twenty-nine valid cases were recorded on the revised sign-out/return sheet. The time to complete a simple laceration repair, using the supply cart, varied from 10 minutes to 116 minutes, with a mean of 36.9 minutes (SD=25.0), a statistically significant average decrease (p = 0.005) of 31.4 minutes.

**Conclusion:**

The use of a suture repair cart in the ED reduced the time required for physicians to perform a simple laceration repair. A minimal 2-cycle PDSA/PS QI process allowed residents and staff to participate in a quality-improvement project, even in a potential staffing-shortage environment.

## INTRODUCTION

A letter to President Biden, dated 7 November 2022 and signed by 35 national health agencies and physician organizations, emphasized that unprecedented and rising staffing shortages throughout the health care system have brought the issue of emergency department (ED) boarding, with its negative impact on patient care, to a crisis point.^1^ ED boarding issues (holding admitted patients in the ED, while waiting for an inpatient bed to open) occur when inpatient facilities are full or under-staffed.

Currently, over half of the hospitals responding to a Nursing Solutions Inc 2023 survey reported a registered nurse vacancy rate of greater than 15%.^2^ The nursing shortage, and its impact on ED care, is not a recent development.^2–6^ In 2001, the United States Government Accountability Office issued a report outlining multiple factors impacting the nursing shortages (e.g., aging workforce, job dissatisfaction, heavy workloads, increased overtime, inadequate staffing, insufficient support staff).^6^ In a recent online posting for Nurse Journal, Morris emphasized the emergency room nursing shortage “has a palpable effect on patient outcomes, nurse burnout rates, and staff retention.”^3^

Staffing shortages, whether due to unfilled positions or staff calling in sick due to burnout or other reasons, can negatively impact an organization’s ability to implement and complete quality-improvement (QI) projects.^7,8^ Often, the unfortunate side-effect of this problem is institutional operational failures, defined as a “breakdowns in system processes that hinder care, erode quality, and threaten patient safety.”^7^ Most hospital operational failures involve equipment and supplies.^9,10^

For example, laceration repairs account for a significant portion of ED visits. In 2021, the Centers for Disease Control and Prevention estimated that cuts or piercings accounted for approximately 2,002,000 ED visits per year (see Table 16 in the report: All injury visits, Cut or pierce).^11^ Inefficient simple laceration repair (defined as a superficial laceration measuring < 5.0 cm in length and not requiring multi-layered closure) may significantly increase the time that patients spend in an ED.

An ED resident in a community-based hospital observed that the time needed to gather laceration-repair supplies from multiple locations was negatively impacting timely patient care and provider efficiency. Given the benefits of procedure-designated supply carts for plastic-surgery procedures, cardiac-arrest procedures, and multiple-phase procedures have been established,^12–15^ the resident suggested conducting a PDSA/PS QI project, to explore the feasibility of having a suture repair cart in the ED, located near patient-care bays.

Aims Statement: The primary aims for this project aligned with the Institute of Medicine’s six aims for quality health-care system (see Table 1).^16^ They were two-fold: 1) to explore the time-to-repair benefits of having a laceration-repair supply cart available in the ED and 2) to determine the feasibility of conducting a simple multi-cycle PDSA/PS QI project in a busy clinical environment that has a potential for experiencing chronic staffing shortages (e.g., unfilled positions, absent staff). A secondary aim was to provide the ED residents opportunities to complete a residency requirement for scholarly activity according to the American College of Graduate Medical Education by “systematically analyzing practice using quality improvement methods and implementing changes with the goal of practice improvement,” focusing on the benefits of implementing a multi-cycle PDSA project.^17^

**Table 1.** Crossing the Quality Chasm – The Institute of Medicine’s Six Aims for a Quality Healthcare System^16^

1	**Safe**: avoid injuries to patients (e.g., medical errors)
2	**Timely**: reduce wait times and delays in receiving care
3	**Efficient**: avoid waste (e.g., not repeating tests unnecessarily)
4	**Effective**: practice evidence-based medicine
5	**Equitable**: provide high-quality care to all, regardless of demographics, geographical location, socio-economic status, etc.
6	**Patient-Centered**: ensure that patient values guide all clinical decisions

## METHODS

*Background.* A traditional hunt-and-gather method was used for obtaining laceration-repair supplies in the ED. The laceration repair kit contained ten 4x4 gauzes, five 2x2 gauzes, two medicine cups, one scissors, one needle holder, one forceps, one 18-gauge needle, one 25-gauge needle, one 10-ml syringe, one fenestrated drape, one regular drape, and one towel. The kit was located in the trauma bay storage rack. When a laceration repair was needed, staff often had to collect additional supplies (e.g., extra gauze pads, sutures, bandaging, sterile saline or water, betadine or surface antiseptics, sterile gloves, and basin) from two ED supply rooms and the Omnicell^®^.

To decrease the time involved with the hunt-and-gather method, an ED resident examined alternative methods to access the needed supplies. The motivation to decrease response time to complete a procedure was quality of patient care. Improved procedures can affect, directly or indirectly, (a) patient safety, (b) patient satisfaction, (c) resident’s use of time in direct patient care, and (d) timely door-to-disposition of patients. A multidisciplinary team was created to evaluate the options for addressing this time-consuming supply-gathering process. The project team consisted of an ED faculty member, the ED resident, and an ED nurse manager. The team opted to implement a laceration-repair supply cart in the ED.

*Study design.* This project was determined by the hospital Institutional Review Board to be non-human-subject research and was approved by the hospital Scholarly Activity Review Committee. A Plan-Do-Study-Act theoretical framework, as outlined by Taylor and colleagues,^18^ was implemented. A 2-cycle process was outlined; steps for completion were deliberately kept simple so that the project could be completed in a timely manner, regardless of staffing levels.

*Setting*. This project was implemented in a community-based hospital ED with an average of 46,000 visits per year. Simple laceration repair procedures for adults and children of all ages were the basis for data collection. ED faculty members and residents have privileges to repair simple lacerations in the ED. Usually, two staff members (i.e., a resident and either a nurse or technician) participate in the procedure.

*Procedure*. Two PDSA/PS QI cycles were planned: Cycle 1 - to collect base-line data using a hunt-and-gather supply process, annotated as (a) time of laceration-repair kit sign-out, noted on a sign-out sheet next to the kit in the storage location, and (b) time of kit return, noted on a separate sheet in the same storage location; Cycle 2 - to measure time to conduct a laceration repair using a specialty supply cart, with sign-out and return time sheets located on the cart. Resident participation in the project was requested at weekly resident-education meetings.

## RESULTS

*Cycle 1:* Cycle 1 of the 2-cycle PDSA process was planned as documented below (Table 2). The sign-out/return time sheets contained no resident or patient information or identifiers.

**Table 2.** Cycle 1 PDSA/PS QI Process Baseline Documentation

Plan	Objective: To collect baseline data for laceration-repair procedure completion times. Who: ED residents. Residents will be asked to participate in the baseline data collection at the weekly resident education meetings. What: Laceration repair kit sign-out and return sheets, located near the laceration-repair kit, will be used to collect baseline data. They will contain date and time columns for sign-out and return. When: A 7-week data collection period will be used. Where: Laceration-repair kit supply storage rack.
Do	The plan was implemented as outlined. Problems and Unexpected Observations included: Staff forgetting to document sign-out and/or return times
Study	Summary statistics were calculated (see Statistical Results below). Of the seventeen recorded sign-out times, only twelve had a corresponding documented return time. A review of barriers to collecting accurate sign out/return times was conducted via conversations with residents and staff. The predominant barriers identified were (1) location of the sign-out/return sheets, (2) separate sheets for sign-out and return recordings, and (3) lack of repeated reminders to participate in study documentation.
Act	The steps to implement the procurement, implementation, and restocking of a laceration-repair supply cart were outlined, with a completion goal of 6 weeks: Compilation of a list of supplies needed for the specialty cart Purchase of a cart that could accommodate all the needed supplies Identification of a convenient location for the supply cart Formulation of a hospital policy and procedure for restocking the cart; notify/train staff regarding the restocking process. The modifications needed to improve sign-out and return times were identified: Use only one sheet to document sign-out/return times, attached to the supply cart for easier completion. Notify staff at all weekly and monthly meetings of the availability of the supply cart, its contents, and plans for restocking. Remind residents at meetings of the importance of data collection and the need to complete the sign-out/return form in a timely manner. A second data collection process will be implemented in PDSA Cycle 2, using the modifications listed above, upon procurement and installation of the supply cart and applicable hospital stocking policies, as well as completion of training for appropriate cart-maintenance staff.

**Figure 1.**
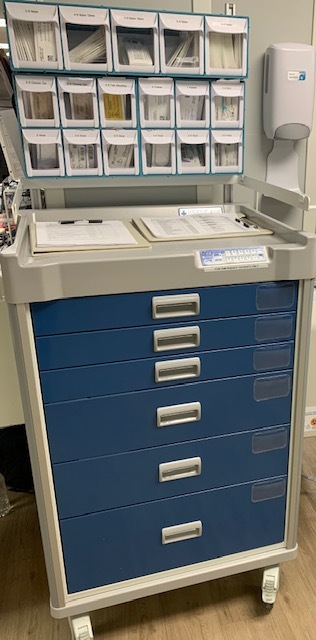
The Simple-Laceration Supply Cart

*Cycle 2*: The ACT phase of Cycle 1 was completed within the planned time-frame of six weeks. The list of supplies developed by the project team influenced the cart-purchase process (see Table 4 for the list of supplies; see Figure 1 for a picture of the cart). The cart has a top rack with three tiers consisting of 17 drawers for holding sutures and a base consisting of six supply drawers. For easy access and return, the cart is positioned outside the nursing supply/tube station room, opposite the trauma bays. The hospital staff responsible for restocking the cart between procedures were identified and trained in accordance with hospital policies.

During weekly resident education meetings and monthly department meetings, the ED faculty, residents, and staff were instructed about the availability of the cart for use in simple laceration repairs, its contents, and the procedures for restocking. Then, Cycle 2 was implemented as documented below (Table 3). The sign-out/return sheet did not contain any resident or patient information or identifiers and was attached to the supply cart as planned.

**Table 3.** Cycle 2 PDSA/PS QI Process Documentation

Plan	Objective: To collect data for laceration-repair procedure completion times using a simple-laceration supply cart. After the procurement, stocking, and restocking-procedure training and implementation of a laceration-repair supply cart, in addition to the notification and training of staff regarding the cart, the following will be completed: Who: ED residents. At the weekly education meetings, residents will be asked to participate in the follow-up data collection. What: A sign-out/return sheet, located on the supply cart, will be used for collection of baseline data. The sheet will contain a date and time column for sign-out and time column for return. When: A 7-week data collection period will be used. Where: Cart will be located outside the nursing supply/tube station room.
Do	The plan was implemented as outlined. Staff forgetting to document sign out or return times continued to be an issue.
Study	Summary statistics were calculated (see Results below). Of the thirty-four recorded sign-out times, twenty-nine had a corresponding documented return time.
Act	After reviewing the results, the project team and the ED staff decided to retain the laceration-repair supply cart. The results of both cycles were shared with the ED and hospital administration, and they were presented at the monthly ED department meeting.

**Table 4.** Simple Laceration-Repair Cart Drawer Supplies

**Drawer 1**	**Drawer 2**
1% lidocaine 20 mL vials lidocaine with epinephrine vials filter needles 3 sizes of injection needles Chloroprep® three sizes of syringes	Four 2x2 gauze packets 12 T-rings 1 box t-strips scalpels (10/11/15/20 blades) staple removers suture removal trays cauterization pens
	
**Drawer 3**	**Drawer 4**
3 sizes of staplers: extra-large, large, small 3 sizes of microMend® Steristrips® skin glue	2 packets of OR towels 2 bottles of iodine 2 bottles of Iodoform® in 2 sizes 12 packs of 4x4s 3 bottles of ethyl chloride 12 boxes 4x4 gauze 10 packs of Telfa®
	
**Drawer 5**	**Drawer 6**
6 Chux^®^ pads 10 Zerowet^®^ 30 saline flushes sterile gloves – 10 each, sizes 6.5-8.0 20 rolls of tape Petrolatum^®^ gauze ADAPTIC^®^ wound dressings 1 box of masks with shields 6 stretch bandage rolls	18 laceration tray kits 2 bottles of hydrogen peroxide 4 1L bottles of normal saline

## Data Collection and Analysis

To estimate the characteristics of the study sample, our statistical approach consisted of means and frequencies. Two-sample Wilcoxon rank-sum (Mann-Whitney) test was conducted to assess the effectiveness of the suture cart implementation. Analyses were performed in Stata 15.1.

There was a total of 12 valid cycle 1 baseline cases recorded on the sign-in/return sheets. The baseline time range to perform simple laceration repair was 26 to 151 minutes, with a mean of 68.3 minutes (SD=40.7). The limited number of recorded cases was due to exclusion of cases lacking a documented end time.

There were 29 valid cycle 2 suture-cart implementation cases recorded. The time range to perform laceration repair utilizing the suture cart was 10 to 116 minutes, with a mean of 36.9 minutes (SD=25.0).

The results suggest there is a statistically significant difference in the treatment time (z = -2.796, p = 0.005). There was an average decrease of 31.4 minutes to perform a simple laceration repair after implementation of the suture cart.

**Table 5.** Treatment Time with and without the Suture Cart

Laceration Repair Time	Without Suture Cart (n = 12)	With Suture Cart (n = 29)	p-value^a^
Shortest repair time (minutes)	26	10	
Longest repair time (minutes)	151	116	
Median	50.5	30	
Average repair time (mean, ± SD)	68.33 (± 40.75)	36.93 (± 24.97)	z = -2.79 p = 0.005

^a^ p-value corresponding to the results of the Wilcoxon rank-sum (Mann-Whitney) test.

Due to the project’s results, the ED administration approved the permanent placement of a suture cart in the ED.

## DISCUSSION

The two purposes of this project were accomplished: The first aim of the 2-cycle PDSA/PS QI project - to investigate the potential benefits associated with the availability of a specialty-supply cart for simple laceration repair in the ED - was proven. Use of the supply cart in the ED significantly decreased the time involved in a simple laceration repair, with an average decrease of 32.4 minutes per repair incident. Prior to implementation of the cart, residents had to obtain supplies from multiple areas of the ED, which caused delays in performing the procedures. Also, residents would often have to leave the patient’s bedside to obtain materials that they had not anticipated needing.

The suture cart contains all the necessary equipment to perform a laceration repair, thus making acquisition of needed material during suture repair efficient and time saving. Since the suture cart is on wheels, it is easily moved to the patient’s location, thereby eliminating the need for the operator to leave the bedside during the procedure.

Efficient procedures in the ED not only have the potential to improve patient satisfaction, the efficiency also permits providers, including physicians, nurses, and technicians, to have more time for other patient care issues. This efficiency may also improve Press Ganey scores, door-to-doctor times, time to disposition, and other metrics tracked in the ED.

The second aim of the project – to determine the feasibility of conducting a simple multi-cycle PDSA/PS QI project in a busy clinical environment that can experience chronic staffing shortages – was accomplished. A key aspect of this 2-cycle PDSA project was its simplicity. Cycle 1 primarily involved planning (an overview of the end goal) and minimal data collection, thereby requiring minimum effort on the part of residents and staff.

The organization for Cycle 2 (procurement of the supply cart and supplies, as well as research and adherence to hospital policies and procedures) involved administrative and support hospital staff, rather than residents; however, the organization process afforded the project-initiating resident an opportunity to experience the nuances of implementing change in a diversified organizational environment, including exposure to logistics, with minimal time and effort. Experience with the administrative aspect of implementing change can inform and simplify additional iterations of any QI project.

Notably, data collection variables (sign out and return times) were minimal. This was intentional. The goal was to complete the project within a reasonable time frame (5-6 months), regardless of staffing levels. Taylor and colleagues suggest starting with small PDSA/PS QI projects. “The pragmatic principles of PDSA cycles promote the use of a small-scale, iterative approach to test interventions…Starting with small-scale tests provides the users with freedom to act and learn; minimizing risk to patients…” (p. 291)^18^

After Cycle 1 and Cycle 2 data were reviewed and during preparation of this report, it became evident that a standardized document for recording project details would have greatly enhanced the ability to record significant information about the project and to address issues in a timely manner. A recommendation was made to develop this document for future PDSA/PS QI projects.

Moreover, ED residents’ participation in quality improvement (QI) projects has the potential to not only improve ED policies and procedures, but also to instill the value of leading and delivering much needed change in the ED. The iterative nature of Plan-Do-Study-Act/Patient Safety (PDSA/PS) QI projects can provide a simplified framework for a project that can be accomplished in a busy setting, thereby allowing ED residents to engage in one or more cycles of a QI project.

It was observed that residents who have been exposed to a variety of health-care venues may be able to suggest simple effort-reducing procedures. Allowing these residents to introduce established, effort-reducing procedures into a new environment, via a multi-cycle PDSA/PS QI project, has the potential to benefit service at all levels (e.g., administration, front-line staff, and patients).

More importantly, keeping the process simple can enhance the project participants’ ability to adhere to the PDSA methodology. Several researchers have noted that in many healthcare settings, where staff are utilizing the PDSA process to implement quality improvement, the fast-paced and often chaotic culture of an ED can make it difficult to (1) adequately engage in and complete all aspects of the cycle (e.g., clear definition of the problem and contributing factors, adequate planning, adequate data collection, communicating what has been learned, etc.), and (2) conduct a thorough assessment of the first cycle to more accurately inform a second (follow-up) process-improvement cycle.^18–20^ The simplicity of this project allowed the project team to adhere to the PDSA methodology, engaging in a multi-cycle process and completing both cycles as outlined by Taylor and colleagues.^18^

Despite the positive features of this study, it had limitations: 1) It was conducted in a single community-based hospital, so the findings may have limited applicability. Future studies might include multiple EDs, various types of lacerations, and various levels of staffing; 2) The number of valid baseline cases was low. However, the need for accurate and complete reporting times was discussed with the residents; and, after analysis of the results, it was determined that having more baseline cases would not have significantly affected the findings; 3) Staffing levels in the ED were not assessed in the context of what would be considered adequate levels for the size of the ED. 4) There was not a clear definition of when the sign-out time for cycle 1 should be recorded (e.g., after all the supplies from the various locations were gathered or when the supply kit was taken from its storing location). 5) The sign-out/return sheet lacked a space for comments about what might have adversely impacted the recording time (e.g., inability to return kit/cart after immediately after the procedure due to addressing an urgent patient-care situation).

## CONCLUSION

The utilization of a suture-repair cart in the emergency department of a busy community hospital with limited support staff enabled shorter and more efficient emergency department visits for the repair of simple lacerations, as evidenced by the average 31-minute decrease in time to perform a simple laceration repair. Through the use of a relatively simple 2-cycle PDSA/PS QI project, staff were able to adequately participate in the process, despite staffing levels; and ED residents could complete an American College of Graduate Medical Education requirement for scholarly activity.

### Conflict of Interest

None
